# Sindbis Virus Replicon-Based SARS-CoV-2 and Dengue Combined Vaccine Candidates Elicit Immune Responses and Provide Protective Immunity in Mice

**DOI:** 10.3390/vaccines12111292

**Published:** 2024-11-19

**Authors:** Yihan Zhu, Wenfeng He, Rui Hu, Xiahua Liu, Mengzhu Li, Yuan Liu

**Affiliations:** State Key Laboratory of Virology, College of Life Sciences, Wuhan University, Wuhan 430072, China; 2022102040017@whu.edu.cn (Y.Z.); 2023102040019@whu.edu.cn (W.H.); 2022102040019@whu.edu.cn (R.H.); 2021202040057@whu.edu.cn (X.L.); 2021202040042@whu.edu.cn (M.L.)

**Keywords:** Sindbis virus replicon, SARS-CoV-2 vaccine, dengue virus vaccine, neutralizing antibody, immune response

## Abstract

**Background/Objectives**: Since its emergence in 2019, the rapid spread of SARS-CoV-2 led to the global pandemic. Recent large-scale dengue fever outbreaks overlapped with the COVID-19 pandemic, leading to increased cases of co-infection and posing severe public health risks. Accordingly, the development of effective combined SARS-CoV-2 and dengue virus (DENV) vaccines is necessary to control the spread and prevalence of both viruses. **Methods**: In this study, we designed Sindbis virus (SINV) replicon-based SARS-CoV-2 and DENV chimeric vaccines using two delivery strategies: DNA-launched self-replicating RNA replicon (DREP) and viral replicon particle (VRP) systems. **Results**: Cellular and animal experiments confirmed that the vaccines effectively produced viral proteins and elicited strong immunogenicity. These vaccines induced robust immune responses and neutralizing activity against live SARS-CoV-2, DENV1, and DENV2 viruses. In addition, passively transferred sera from BALB/c mice immunized with these vaccines into AG129 mice provided significant protection against lethal DENV2 challenge. The transferred sera protected the mice from physical symptoms, reduced viral loads in the kidney, spleen, liver, and intestine, and prevented DENV2-induced vascular leakage in these tissues. **Conclusions**: Therefore, combined vaccines based on the SINV replicon system are promising candidates for pandemic control. These results lay a foundation for further development of a safe and effective combination vaccine against SARS-CoV-2 and DENV.

## 1. Introduction

The COVID-19 pandemic has had a severe global impact and, as of July 2024, the World Health Organization reported 776 million cases and 7.1 million deaths worldwide [1]. Dengue fever is a mosquito-borne viral disease caused by dengue virus (DENV) serotypes 1–4 that has an increasing global impact [2]. In dengue outbreaks, DENV1 and DENV2 are the most common serotypes [3,4,5]. While most infections cause mild flu-like symptoms, DENV2 is occasionally linked to more severe cases, including fatal outcomes [3,4,5,6]. It is considered the most dangerous serotype, capable of leading to dengue hemorrhagic fever and dengue shock syndrome [2,6]. Although dengue fever was historically confined to tropical and subtropical regions, in recent years, rising temperatures and shifts in rainfall patterns due to global climate change have allowed dengue-carrying mosquitoes, which include *Aedes aegypti* and *Aedes albopictus*, to invade more countries and regions [7,8,9]. This expansion has led to the spread of dengue fever into areas previously considered low-risk or unaffected, such as Europe and North America, with potential for global dissemination [9,10,11,12]. From 2000 to 2019, global dengue cases surged from 500,000 to 5.2 million [9,13]. In 2023, the spread of dengue in the Americas reached unprecedented levels, with over 4 million cases reported in South and Central America [10,13]. Local transmission was also recorded in Florida, Texas, and Arizona in the United States [10]. Meanwhile, Brazil reported the spread of dengue into its colder southern regions for the first time, and Aedes aegypti was detected in Mexico City [10,14]. Additionally, dengue transmission in southern Europe, particularly in Italy and France, continues to rise [11].

The World Health Organization predicts that over 40% of the global population is at risk of dengue infection, and as global warming progresses, dengue may become endemic in parts of the United States and Europe [13,15]. Therefore, dengue fever could potentially develop into a global pandemic similar to SARS-CoV-2, with significant overlap with regions affected by SARS-CoV-2, resulting in a high risk of co-infection [16,17]. For instance, Pakistan reported 3442 cases of dengue fever during the SARS-CoV-2 pandemic in 2020, with co-infections exacerbating the healthcare crisis [18,19]. Similar co-infections have been reported in South Africa and South America, complicating the diagnosis owing to overlapping symptoms such as fever and headache [20,21]. Therefore, developing vaccines that can simultaneously control both SARS-CoV-2 and dengue fever is crucial. Such vaccines may help strengthen global health security because of improved vaccination efficiency, reduced doses and costs, and the ability to manage overlapping outbreaks.

A study involving DENV-seropositive children in India confirmed that SARS-CoV-2 IgG antibodies could alleviate the symptoms of dengue fever [22,23]. Another study reported that children infected with SARS-CoV-2 exhibited significantly reduced symptoms and no fatalities on subsequent infection with DENV [17]. Computational modeling further demonstrated that antibodies targeting the DENV E protein could bind to the SARS-CoV-2 receptor-binding domain (RBD) of the S1 subunit of the spike protein region, thereby blocking its interaction with the human angiotensin-converting enzyme 2 receptor [24,25,26]. Furthermore, the serum of patients with SARS-CoV-2 was reported to neutralize the DENV2 virus at high dilutions [27]. Moreover, administration of anti-S1-RBD IgG antibodies to mice significantly reduced hemorrhage and decreased serum NS1 levels following DENV infection [27]. Collectively, these studies suggest partial cross-protection between the two RNA viruses [28], indicating a combined SARS-CoV-2 and DENV vaccine as a feasible and promising option.

Significant progress has been made in the development of novel SARS-CoV-2 vaccines. Vaccines using the SARS-CoV-2 Spike S1 RBD as the primary immunogen have been widely administered globally to effectively curb disease spread [29,30,31]. However, other new SARS-CoV-2 variants, such as Omicron and Delta, have also emerged [32]. The conserved functional domains heptad repeat 1 and 2 (HR1 and HR2) of the spike proteins elicit broadly neutralizing antibodies and strong immune responses in various animals and are novel promising immunogenic targets [33,34]. Vaccines using these proteins provide nearly complete protection against SARS-CoV-2 infection in both mouse and non-human primate models [35,36]. Nevertheless, the development of effective dengue vaccines is challenging. Although some vaccines are currently undergoing clinical trials, safe and effective vaccines to control dengue outbreaks remain unavailable [37]. Dengvaxia (CYD-TDV), the only approved dengue vaccine, is a live-attenuated vaccine that uses the yellow fever virus genome as the backbone, with the PrM and E coding sequences replaced with those of DENV [38]. However, this vaccine may increase the risk of severe dengue and hospitalization in children who never had dengue, who acquire infection after vaccination owing to antibody-dependent enhancement [39]. Therefore, the effectiveness of this vaccine is restricted to individuals who have previously contracted dengue and reside in dengue-endemic regions [37,38,39]. Additionally, evaluations of the Dengvaxia vaccine show an efficacy rate of 50–80% against DENV1, DENV3, and DENV4, but only 35–42% against DENV2 [40,41]. Therefore, a safe and effective dengue vaccine, particularly for DENV2, remains critically needed. Novel approaches may include antibodies targeting structural protein E of DENV, particularly 80% of the envelope protein (80E) fragment, which can prevent viral attachment and entry [42]. Furthermore, vaccination with the nonstructural protein NS1 can generate antibodies that reduce viral replication without causing ADE [43,44]. Therefore, 80E and NS1 are promising candidates for dengue vaccine development.

The choice of the delivery platform is critical for vaccine development. Alphavirus-based DNA-launched self-replicating RNA replicon vaccines (DREP) exhibit good stability and can be stored at room temperature for long periods without requiring freezing during storage and transport [45], thus conferring significant advantages over conventional vaccines in combating global pandemic outbreaks. Sindbis virus (SINV)-based DREP vaccines activate innate and adaptive immune responses and effectively induce virus-neutralizing antibody production [46,47]. In addition, the viral replicon particle delivery system (VRP) replaces the coding sequences of the viral structural proteins with those of foreign target proteins, producing a self-amplifying RNA that enables extensive translation of the corresponding antigens [48,49]. Replicon RNA lacks viral structural genes and requires helper plasmid co-transfection for effective packaging, rendering it replication-deficient and thus safe for large-scale vaccine production [50]. Furthermore, the DREP and VRP systems can encode any target antigen and facilitate consistent upstream supply chains and downstream processes for each vaccine product, thus allowing rapid vaccine development and deployment.

This study aimed to develop SINV-based DREP and VRP vaccines encoding antigens from both SARS-CoV-2 and DENV. We evaluated the immune responses and neutralization capacity induced by these vaccines against live SARS-CoV-2, DENV1, and DENV2 viruses. Furthermore, through passive transfer experiments, we investigated the protective effects of the sera against DENV2 infection in AG129 mice. Our results showed that the combined vaccine delivered by both methods effectively activated the immune system of mice, induced antibody production against both SARS-CoV-2 and DENV, and provided protection against DENV2.

## 2. Materials and Methods

### 2.1. Cells and Viruses

BHK-21 and Vero E6 cells were purchased from ATCC and maintained in DMEM (Gibco, Waltham, MA, USA, 11995065) supplemented with 10% fetal bovine serum (Gibco, A3160902), 1% L-glutamine (Gibco, 25030081), and 1% penicillin–streptomycin (Gibco, 15140122) at 37 °C in a humidified CO_2_ incubator with 5% CO_2_. C6/36 cells, also purchased from ATCC, were maintained in L-15 medium (Gibco, 11415064) supplemented with 10% FBS, 1% L-glutamine, and 1% penicillin–streptomycin at 28 °C in a humidified incubator without CO_2_. All the experiments with infectious virus were performed following standard operating procedures in biosafety level (BSL)-3 (SARS-CoV-2 infection) and BSL-2 (DENV infection) facilities and approved by the Biosafety Committee of the State Key Laboratory of Virology, Wuhan University. The SARS-CoV-2 Omicron BA.1 strain (strain number HB0000428) was provided by the Hubei Provincial Centre for Disease Control and Prevention and was propagated and titrated in Vero E6 cells. The DENV serotype 1 strain GDV155 was obtained from the China Center for Type Culture Collection (CCTCC, Wuhan, China), propagated in C6/36 cells and titrated in Vero E6 cells. The DENV serotype 2 TSV01 strain was generously provided by Dr. Bo Zhang (Wuhan Institution of Virology, Chinese Academy of Sciences), propagated in C6/36 cells and titrated in Vero E6 cells.

### 2.2. Construction of SINV Replicons with Target Antigen Combinations

SINV replicons were constructed using a full-length SINV (HRsp strain) clone in a pcDNA3.1 vector using standard cloning techniques. The replicon was modified with a CMV promoter/enhancer and a T7 polymerase promoter. The structural genes of SINV were replaced with genes encoding foreign antigens. The antigen sequences utilized were derived from the RBD and the HR1-HR2 regions of the SARS-CoV-2 spike protein, as well as the NS1 and the 80E protein of DENV1 and DENV2. The SARS-CoV-2 and dengue antigen sequences were ligated using overlap PCR and inserted into the SINV replicon via PmeI and PacI. The gp67 signal peptide sequence was incorporated at the N-terminus of the foreign antigen genes to facilitate protein secretion, while the 3×FLAG tag sequence was appended to the C-terminus of the foreign antigen sequences to enable detection. Subsequently, the replicon plasmid was ligated to poly(A) followed by a hepatitis delta virus (HDV) ribozyme sequence. The SINV helper plasmid was constructed based on the DHBB plasmid and incorporated into a pcDNA3.1 vector [51].

### 2.3. Production of SINV Replicon Particles

To produce VRPs, BHK-21 cells were transfected with in vitro-transcribed RNAs from the replicon and DHBB helper plasmid. Supernatants were harvested 48 h later, centrifuged for 15 min at 1200× *g*, and frozen at −80 °C in small aliquots. The titers of the replicon particles were determined by plaque assay in Vero E6 cells.

### 2.4. Western Blotting

BHK-21 cells, after being transfected with SINV-replicons, were lysed using RIPA Lysis Buffer (Beyotime Biotechnology, Shanghai, China, P0013C) supplemented with a protease inhibitor cocktail (Sigma-Aldrich, St. Louis, MO, USA, P7626). The lysates were then placed on ice for 15 min. Following this, the lysates were centrifuged at 13,000× *g* for 15 min. The supernatants were collected and mixed with 5× loading buffer (Beyotime Biotechnology, P0015L), followed by heating to 100 °C for 10 min to denature the proteins. The sample was then resolved on an SDS-PAGE gel and transferred onto polyvinylidene difluoride (PVDF) membranes for further analysis. To block non-specific binding, the PVDF membranes were incubated with 5% non-fat milk in TBST for 1 h. Next, the PVDF membranes were incubated with primary antibodies—rabbit anti-FLAG (1:2000; Cell Signaling, Danvers, MA, USA, 14793T) and mouse anti-GAPDH (1:3000; Proteintech, Wuhan, China, 60004-1-Ig)—incubated at 4 °C overnight. Following three washes with TBST, the membranes were incubated with Peroxidase AffiniPure Goat Anti-Mouse IgG (H + L) (1:5000; Jackson ImmunoResearch, West Grove, PA, USA, 115-035) and Peroxidase AffiniPure Goat Anti-Rabbit IgG (H + L) (1:5000; Jackson ImmunoResearch, 111-035-003) for two hours at room temperature. Finally, the membranes were washed three times with TBST to prepare them for subsequent detection and analysis.

### 2.5. Immunofluorescence and Confocal Microscopy

In the SARS-CoV-2 neutralization assay, BHK-21 cells transfected with SINV-replicon plasmids or infected with SINV replicon particles, as well as Vero E6 cells plated in 24-well dishes, were washed three times with 0.3% Triton X-100 PBS for 10 min each. Subsequently, the cells were fixed in 4% formaldehyde for 30 min. Following fixation, the cells were blocked in Blocking Buffer (Beyotime Biotechnology, Shanghai, China, P0102) for 45 min. They were then incubated with primary antibodies diluted in blocking buffer at 4 °C overnight. The primary antibodies used were rabbit anti-FLAG (1:1000 dilution, Cell Signaling, Danvers, MA, USA, 14793T) or anti-SARS-CoV-2 nucleocapsid antibody (kindly provided by Dr. Huan Yan at Wuhan University) at a 1:1 dilution. After three additional washes, the cells were incubated with secondary antibody and DAPI for 2 h at room temperature. The secondary antibodies (Alexa 488, Invitrogen, Waltham, MA, USA) were used at a 1:1000 dilution, while DAPI was used at a concentration of 1 μg/mL. The samples were mounted in Vectashield Mounting Media (Vector Laboratories, Burlingame, CA, USA) and visualized using a Leica SP8 confocal laser scanning microscope.

### 2.6. RT-qPCR

Total RNA was extracted from mouse tissues or cells using TRIzol reagent (Invitrogen, 15596018CN) according to the manufacturer’s protocol. First-strand cDNA was synthesized using the HiScript III RT SuperMix Reverse Transcription Kit (Vazyme, Nanjing, China, R323-01). Real-time PCR was performed for each sample using SYBR Green (Vazyme, Q311-02) on a QuantStudio 6 System (Thermo Fisher Scientific, Waltham, MA, USA). The relative abundance of gene transcripts was normalized to GAPDH as an endogenous control using the 2^−ΔΔCT^ method.

### 2.7. Animal Immunizations

All animal experiments were approved by the Institutional Animal Care and Use Committee of Wuhan University (protocol number SKLV-AE2022005) and performed in accordance with the relevant guidelines and regulations. BALB/c mice were bred and housed in specific pathogen free (SPF) animal facility at Wuhan University.

The DREP vaccine was administered via intramuscular injection (100 μg each time), and the VRP vaccine was administered via tail vein injection (10^6^ PFU each time). To maximize the immune response in the mouse model, the mice were immunized on days 0, 7, and 14. The animals were weighed at the time of arrival and subsequently at seven-day intervals, and they were monitored daily for any signs of discomfort post-vaccination. Sera were collected on days 0 and 21 by retro-orbital puncture and then analyzed using enzyme-linked immunosorbent assay (ELISA).

### 2.8. ELISA Detection of SARS-CoV-2 S Protein IgG Antibodies in the Sera

The SARS-CoV-2 Omicron Spike protein IgG detection kit (Vazyme Biotech, DD3208) was used to assess serum IgG reactivity against the SARS-CoV-2 Omicron S protein. These ELISA detection tests were performed according to the manufacturer’s instructions. Briefly, sera were added to the appropriate wells and incubated at 37 °C for 1 h. Positive and negative control wells were included on each plate. The plates were washed three times before incubation with the HRP-conjugated IgG for 30 min at 37 °C. The final washing steps were repeated, followed by incubation with 100 µL/well Super Sensitive TMB for 10 min at 37 °C. The reactions were stopped by the addition of 50 µL/well Stop Solution. HRP catalyzes the color development of the substrate, and the absorbance at 450 nm was measured within 30 min using a microplate reader. The absorbance value is positively correlated with the concentration of IgG to be detected.

### 2.9. ELISA Detection of SARS-CoV-2 S Neutralizing Antibodies in the Sera

The level of neutralizing antibodies in the sera was tested using the SARS-CoV-2 Omicron variant S protein neutralizing antibody ELISA test kit (Friendbio Science & Technology, Wuhan, China, EKn-Cov003), which is based on the reactivity of neutralizing antibodies against the SARS-CoV-2 S protein. The immobilized S-protein RBD on the pre-coated plate can be specifically bound to HRP-ACE2, and then the color is developed by TMB, catalyzed by the HRP enzyme of HRP-ACE2. If the sample to be tested or the standard contains neutralizing antibody against the S protein, it will prevent the HRP-ACE2 coupler from binding to the immobilized RBD fragment, so the neutralizing antibody content is negatively correlated with the TMB color development value. Negative control, positive control, and samples to be tested were added to the plate, then the HRP working solution was added to each well, gently mixed, and incubated at 37 °C for 60 min; the plates were washed three times. Then, 100 μL of TMB substrate solution was added to each well and left at room temperature for 15 min, then 50 μL of substrate reaction termination solution was added to each well, followed by the measurement of the absorbance values at 450 nm. The inhibition rate is used to determine the amount of anti-SARS-CoV-2 neutralizing antibody in the sample. The inhibition rate% = (negative control of the kit OD450 − sample OD450)/control OD450 × 100%.

### 2.10. SARS-CoV-2, DENV1 and DENV2, and SINV Neutralization Assays

Briefly, sera were serially diluted in DMEM and incubated with 200 TCID50 SARS-CoV-2, 20,000 TCID50 DENV1 and DENV2, or 2000 TCID50 SINV for 30 min at 37 °C. The mixture was then infected with Vero E6 cells in 24-well plates. For SARS-CoV-2 and SINV, the plates were incubated for 24 h at 37 °C in 5% CO_2_ in humidified CO_2_ incubators before RT-qPCR and immunofluorescence assays. For DENV1 and DENV2, the plates were incubated for 3 days prior to performing the plaque assay.

### 2.11. In Vivo Determination of Vaccine Potency: Passive Transfer Method

AG129 mice were generously provided by Dr. Bo Zhang (Wuhan Institute of Virology, Chinese Academy of Sciences) and were housed in pathogen-free ABSL-2 animal facilities at Wuhan University. All animal infection experiments were conducted in accordance with standard operating procedures in the ABSL-2 facilities, with approval from the Biosafety Committee of the State Key Laboratory of Virology, Wuhan University (protocol number SKLV-G2024-053). AG129 mice (6–8 weeks old) were divided into three groups (n = 6 per group). In the experimental groups, mice were injected intravenously with 100 μL of sera from BALB/c mice immunized with either VRP RBD-DENV2-80E or HR1HR2-DENV2-NS1 vaccines. In the control group, mice were injected intravenously with serum from BALB/c mice injected with SINV replicon virus particles only, followed by intravenous injection of a sublethal dose of DENV-2 TSV01 (10^4^ PFU/mouse) 2 h later. Mouse survival, body weight, and clinical signs were monitored daily for 7 days. Clinical signs were scored: 0.5, mildly matted fur; 1.0, matted fur; 1.5, fur loss; 2.0, dorsal curling; 2.5, limited movement/paralysis of one leg; 3.0, no movement on stimulation/paralysis of hind legs; and 4.0, death or euthanasia.

### 2.12. Measurement of DENV-Induced Vascular Leakage in Tissues of AG129 Mice

Vascular leakage was evaluated using Evans blue dye. Briefly, on day 5 post-DENV2 infection, 0.2 mL of 0.5% Evans blue solution was injected into the tail vein of the AG129 mice. Two hours later, the mice were euthanized and perfused with 50 mL of PBS. The livers, kidneys, spleens, and intestine were collected and photographed.

### 2.13. Plaque Assay

For the SINV pseudovirus and the DENV1 and DENV2 plaque assays, the plates were incubated with DMEM containing 10% FBS, 1% L-glutamine, 1% penicillin–streptomycin and 1% methylcellulose (Sigma-Aldrich, M0512) for 3 days at 37 °C in 5% CO_2_ in humidified CO_2_ incubators. Plaques were fixed with 4% formaldehyde, stained with 1% crystal violet (Sigma-Aldrich, C6158), and counted under a microscope.

### 2.14. Quantification and Statistical Analyses

All data were analyzed using a one-way or a two-way ANOVA, followed by Dunnett’s multiple comparison test, except for survival analyses, which were performed using the log-rank (Mantel–Cox) test. The thresholds of statistical significance were defined as follows: * *p* < 0.05, ** *p* < 0.01, *** *p* < 0.001, **** *p* < 0.0001, and ns for non-significance (*p* > 0.05). Statistical analysis was performed using GraphPad Prism 8 and Microsoft Excel. The source data and uncropped Western blot images for all figures are included in the Supplementary Material.

## 3. Results

### 3.1. Design of the SARS-CoV-2 and DENV Combined Vaccine

**Figure 1:**
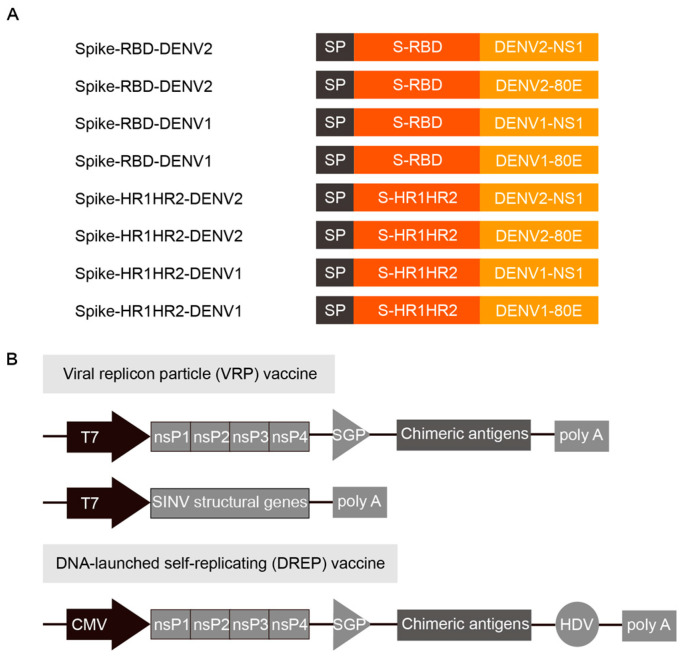
Schematic of the chimeric vaccine antigen design and delivery. (**A**) Antigen combinations for the vaccine. (**B**) Schematic of the vaccine expression system. To target SARS-CoV-2 and DENV, we replaced SINV viral structural genes with genes encoding the corresponding viral proteins. Two methods were used for vaccine production: VRP and DREP vaccines. Abbreviations: RBD, receptor-binding domain; HR, heptad repeat; S, spike; CMV, cytomegalovirus promoter; T7, T7 promoter; SGP, subgenomic promoter; HDV, hepatitis delta virus; poly A, polyadenylation signal; SP, signal peptide.

We designed eight antigen combinations of the SARS-CoV-2 RBD, HR1, and HR2 domains, as well as the DENV1 and DENV2 NS1 and 80E domains, to construct combined vaccines targeting both SARS-CoV-2 and DENV (Figure 1A). Furthermore, we added a GP67 signaling peptide at the N-terminus of the inserted antigen to increase the exposure of the antigen to the immune cells and enhance the immune response by promoting the secretion of foreign proteins on the cell surface. FLAG tag sequences were also added to assess antigen expression using an anti-FLAG tag antibody. Notably, DREP and VRP plasmids were constructed using the SINV replicon as the basic skeleton to develop a simpler and more efficient chimeric vaccine. This system included a replicon plasmid for the coding sequences of the nonstructural proteins nsP1 through nsP4, along with a helper plasmid containing the coding sequences of the structural genes of SINV (Figure 1B), thus enabling chimeric antigen production within the viral replicon particles and ensuring efficient delivery and expression. In addition, a SINV replicon plasmid was used as the control group.

### 3.2. DREP Vaccine Candidates Expressing SARS-CoV-2 and DENV Protein Antigens

**Figure 2:**
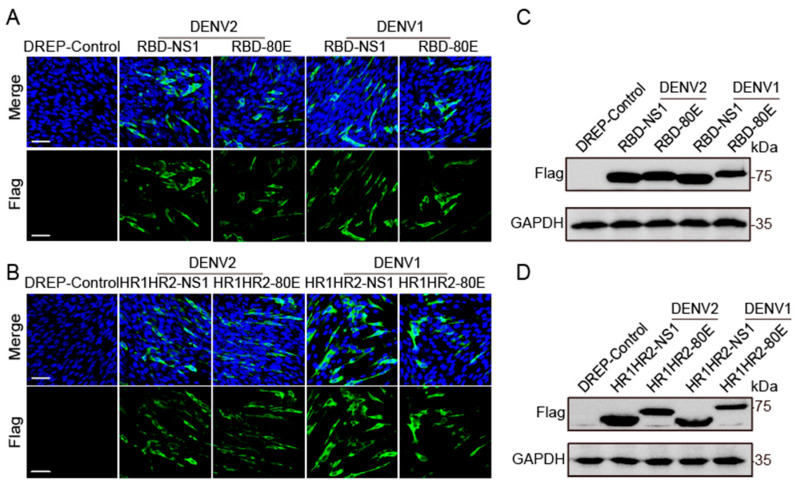
In vitro expression of antigens from the DREP combined vaccine. (**A**,**B**) BHK-21 cells were transfected with DREP constructs containing antigen fragments and immune-stained after 48 h. Antigen expression and localization on the BHK-21 cell surface were observed using fluorescence microscopy (scale bar = 50 µm). Cell nuclei were stained with DAPI (blue), and antigens with FLAG fusion protein tags were stained with a FLAG antibody (green). (**C**,**D**) BHK-21 cells were transfected with DREP constructs containing the conjugated antigen fragments, and cell protein samples were collected after 48 h. Lysates were analyzed using Western blotting with a FLAG-tagged antibody. The controls were transfected with SINV replicon plasmids without antigen segment insert.

We transfected the plasmids encoding SARS-CoV-2 and DENV antigen combinations into BHK-21 cells to test antigen expression. Immunofluorescence staining confirmed successful expression of all eight antigen combinations (Figure 2A,B). Furthermore, as determined using Western blotting, compared with the control groups, the groups expressing RBD-NS1, RBD-80E, HR1HR2-NS1, and HR1HR2-80E antigens showed distinct FLAG signals, confirming their presence (Figure 2C,D). For antigen combinations containing RBD-DENV2-NS1 and HR1HR2-DENV2-NS1, higher expression levels were observed in both immunofluorescence and Western blotting.

### 3.3. RNA Kinetics and Antigen Expression Levels for VRP Vaccine Candidates

**Figure 3:**
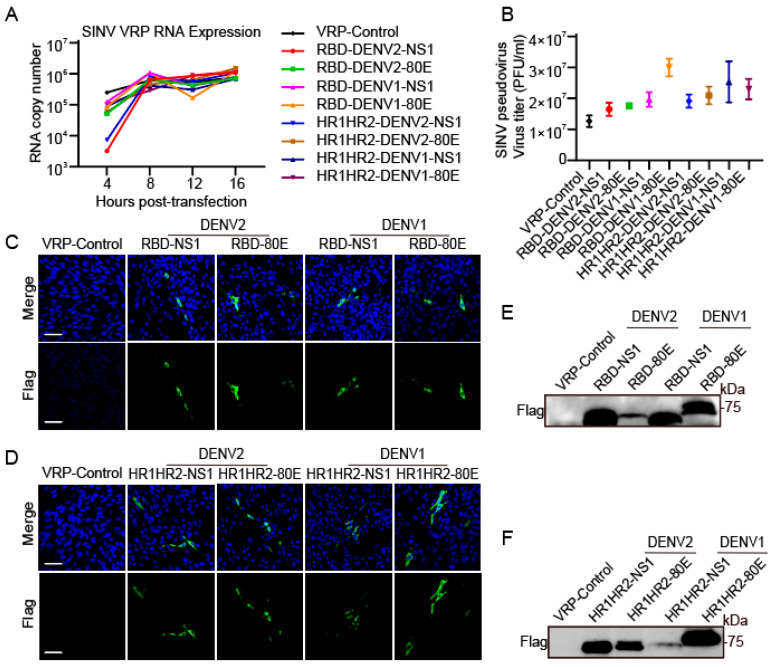
Replication kinetics, viral titers, and in vitro antigen expression of VRP vaccine-infected BHK-21 cells. (**A**) BHK-21 cells were co-transfected with in vitro-transcribed RNAs from a SINV replicon plasmid encoding the vaccine antigen and the helper plasmid expressing SINV structural proteins, and the RNA expression dynamics were examined using RT-qPCR from 4 to 16 h. (**B**) BHK-21 cells were co-transfected with the SINV replicon plasmid encoding the vaccine antigen and the helper plasmid expressing SINV structural proteins, and the supernatant of the cells was collected after 24 h. The titer was determined using a plaque assay, n = 2. Data represent mean ± SD. (**C**,**D**) BHK-21 cells were co-transfected with the SINV replicon plasmid encoding vaccine antigens and helper plasmid expressing SINV structural proteins and immune-stained 24 h later. The antigen expression and localization on the surface of BHK-21 cells were observed using fluorescence microscopy (scale bar = 50 µm). Cell nuclei were stained with DAPI (blue), and antigens were stained with FLAG antibody (green). Antigen expression was detected using immunofluorescence. (**E**,**F**) Antigen expression was assessed using Western blotting. The controls were co-transfected with in vitro-transcribed RNAs from SINV replicon plasmid without antigen insert and a helper plasmid expressing SINV structural proteins.

We also examined the efficacy of the VRP expression system. We co-transfected BHK-21 cells with in vitro-transcribed RNAs from SINV replicon plasmids encoding various antigen fragments and the helper plasmid DHBB, which encodes SINV structural proteins. This process packages replicons into single-round infectious viral particles to deliver antigens to recipient cells. A quantitative analysis revealed high RNA expression from SINV replicon particles at various time points (4, 8, 12, and 16 h post-transfection) for all constructs (Figure 3A). Plaque assays revealed variations in the viral titers of different constructs; however, the PFU of all SINV pseudoviruses were larger than 10^7^, indicating that all of the constructs tested successfully generated packaged replicon particles (Figure 3B). We then collected supernatants from co-transfected cells 48 h post-transfection and incubated them with BHK-21 cells for 24 h to verify viral particle production and antigen expression. The green fluorescence observed in the immunofluorescence assay indicated the successful expression of exogenous proteins in the infected BHK-21 cells (Figure 3C,D). Additionally, Western blot analysis using FLAG antibody confirmed the presence of FLAG-tagged fusion proteins, further validating the expression of the expected antigenic constructs in the VRP vaccine candidates (Figure 3E,F). Collectively, these results suggested that the SINV VRP vaccines could self-replicate and express combinatorial antigens.

### 3.4. Immunogenicity of DREP and VRP Vaccine Candidates in Mice

**Figure 4:**
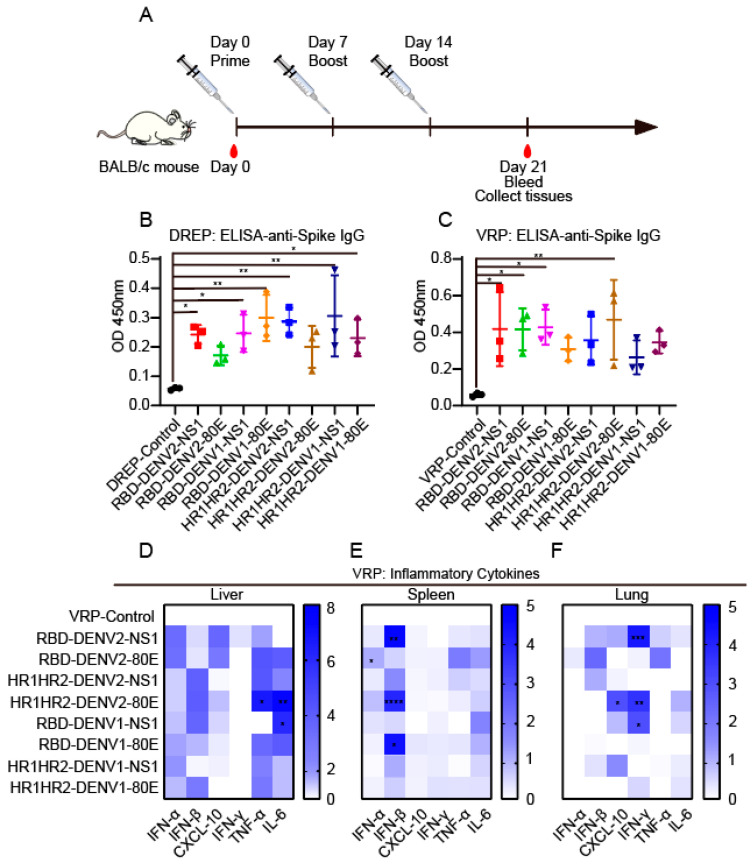
Mouse anti-SARS-CoV-2 IgG antibody and immune responses. (**A**) Schematic of the immunization and sampling process in mice. BALB/c mice (n = 3 per group) were immunized with DREP or VRP vaccines. Mice vaccinated with only SINV replicon plasmids served as DREP vaccine controls; mice vaccinated with only SINV replicon viral particles served as VRP vaccine controls; and mice which received only PBS were used to assess the immunological effects of the SINV replicon delivery vector. Blood samples were collected 21 days post-immunization, and liver, spleen, and lung tissues were also collected. Individual serum samples were assessed using ELISA with plates coated with the SARS-CoV-2 Omicron S antigen. (**B**,**C**) Mouse sera were collected 21 days post-immunization, and IgG antibody levels were determined using ELISA. Levels of serum antibody binding were measured as absorbance (450 nm, n = 3). All experimental groups were compared with the control group. The DREP vaccine control (**B**) was inoculated with SINV replicon plasmids without an antigen insert; the VRP vaccine control (**C**) was inoculated with SINV replicon virus particles only. (**D**–**F**) RT-qPCR assays of IFN-α, IFN-β, IFN-γ, CXCL-10, TNF-α, and IL-6 expression in the liver (**D**), spleen (**E**), and lung (**F**) tissues of the mice immunized with the VRP vaccine for 21 days, n = 3. All experimental groups were compared with a control group that received SINV replicon virus particles. Data are presented as mean ± SD. Each data point represents one individual mouse (**B**,**C**). Statistical analyses were performed using a one-way (**B**,**C**) and a two-way (**D**–**F**) ANOVA followed by Dunnett’s multiple comparison test, respectively. * *p* < 0.05, ** *p* < 0.01, *** *p* < 0.001, and **** *p* < 0.0001.

The mice were immunized with DREP and VRP vaccines based on the SINV replicon system to evaluate the immunogenicity of the combined vaccines. After the initial immunization (day 0), the mice received two booster immunizations on days 7 and 14 (Figure 4A). Serum samples were collected before the initial injection as pre-immune controls. Post-immunization serum samples were collected on day 21 to analyze the IgG antibody levels against SARS-CoV-2 induced by different antigen combinations. All vaccinated mice exhibited seroconversion 21 days post-immunization. ELISA revealed that both SINV DREP and VRP vaccine candidates induced significant antibody titers compared to their control group (Figure 4B,C).

One of the main advantages of the VRP vaccine platform is its ability to induce strong humoral and cellular immune responses. To monitor the immune responses, mice were euthanized 7 days after the third injection to assess the cellular response induced by the combined vaccine. The liver, spleen, and lung tissues were collected to determine inflammatory cytokine levels using RT-qPCR. Compared to the control group, the VRP vaccine group exhibited higher expression of genes encoding inflammatory cytokines in various organs, such as tumor necrosis factor (TNF)-α (HR1HR2-DENV2-80E, *p* = 0.0206) and interleukin (IL)-6 (HR1HR2-DENV2-80E, *p* = 0.0056; RBD-DENV1-NS1, *p* = 0.0462) in the liver, interferon (IFN)-β(HR1HR2-DENV2-80E, *p* = 0.0056; RBD-DENV1-NS1, *p* = 0.0462) in spleen, IFN-γ (RBD-DENV2-NS1, *p* = 0.0005; HR1HR2-DENV2-80E, *p* = 0.0044; RBD-DENV1-NS1, *p* = 0.0349) in lungs, and CXCL-10 (HR1HR2-DENV2-80E, *p* = 0.0378), indicating a strong inflammatory response (Figure 4D–F). These results validated the effectiveness of the SINV DREP and VRP delivery systems in expressing exogenous antigens, demonstrating its potential to elicit strong immune response.

### 3.5. Combined Vaccine Induces Neutralizing Antibodies Against SARS-CoV-2 in Mice

**Figure 5:**
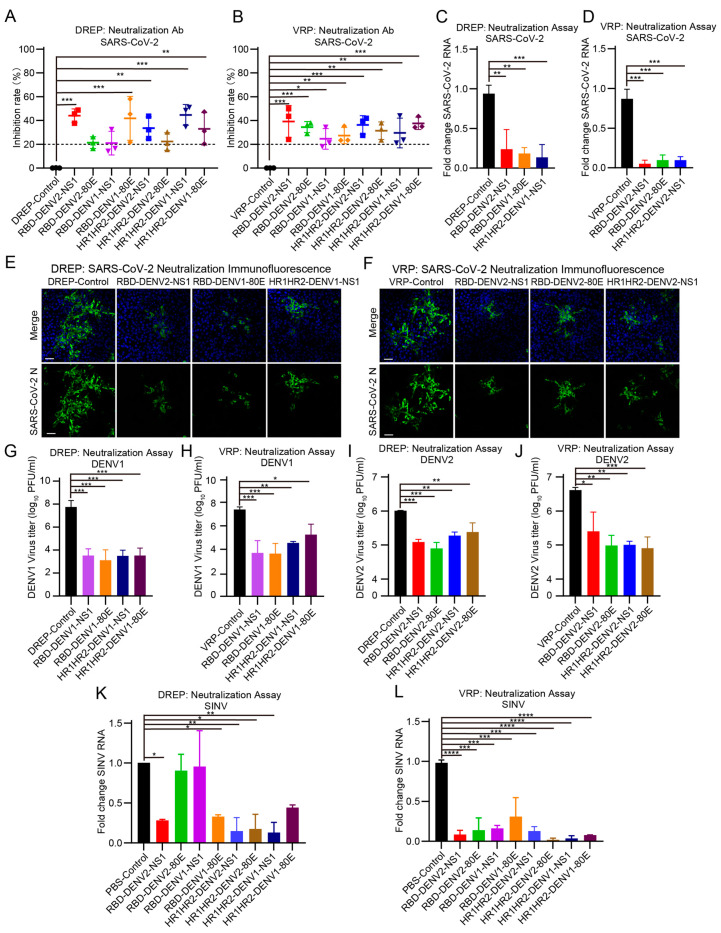
Evaluation of neutralizing antibody responses and authentic virus neutralization assay. (**A**,**B**) Neutralizing antibodies against SARS-CoV-2 in the sera of mice immunized with DREP and VRP vaccines, detected using ELISA, n = 3. (**C**,**D**) Neutralizing effect of sera from mice immunized with DREP vaccines (**C**) and VRP vaccines (**D**) against the SARS-CoV-2 Omicron strain, evaluated using RT-qPCR, n = 3. (**E**,**F**) Neutralizing effect of sera from mice immunized with DREP vaccines (**E**) and VRP vaccines (**F**) against the SARS-CoV-2 Omicron strain, evaluated using an immunofluorescence assay. Green: SARS-CoV-2 nucleocapsid staining; blue: DAPI-stained nuclei. (**G**,**H**) Neutralizing effect of sera from mice immunized with DREP vaccines (**G**) and VRP vaccines (**H**) against DENV1, evaluated using a plaque assay, n = 3. DREP vaccine controls (**A**,**C**,**E**,**G**,**I**) were inoculated with SINV replicon plasmids without antigen insert; VRP vaccine controls (**B**,**D**,**F**,**H**,**J**) were inoculated with SINV replicon virus particles only. (**I**,**J**) Neutralizing effect of sera from mice immunized with DREP vaccines (**I**) and VRP vaccines (**J**) against DENV2, evaluated using a plaque assay, n = 3. (**K**,**L**) Neutralizing effect of sera from mice immunized with DREP vaccines (**K**) and VRP vaccines (**L**) against SINV, evaluated using RT-qPCR, n = 2. The control group received only PBS in the SINV neutralization assay (**K**,**L**). Data are presented as mean ± SD. The scale bars in **E**,**F** are 50 μm. All experimental groups were compared with the control group. Statistical analyses were performed using one-way ANOVA followed by Dunnett’s multiple comparison test (**A**–**D**,**G**–**L**). * *p* < 0.05, ** *p* < 0.01, *** *p* < 0.001, and **** *p* < 0.0001.

To investigate the role of the antibodies produced by the DREP and VRP systems in virus neutralization, the neutralizing antibody titers in the serum of immunized mice were quantified using an ELISA kit specific for SARS-CoV-2 S protein-neutralizing antibodies. A neutralization rate >20% was considered indicative of the presence of neutralizing antibodies. Both vaccine delivery methods effectively stimulated antibody production in mice compared with the levels in the control group (Figure 5A,B). Consequently, we selected the following six antigen combinations for an authentic virus neutralization assay: RBD-DENV2-NS1, RBD-DENV1-80E, and HR1HR2-DENV1-NS1 from the DREP vaccine group and RBD-DENV2-NS1, RBD-DENV2-80E, and HR1HR2-DENV2-NS1 from the VRP vaccine group.

### 3.6. Protective Effect of the Combined Vaccine Against SARS-CoV-2 Viruses

We conducted a live virus neutralization assay using the SARS-CoV-2 Omicron variant (BA.1) to further validate the neutralizing antibody titers in mice. The SARS-CoV-2 strain was pre-incubated with mouse serum and used to infect Vero E6 cells. RT-qPCR results showed a significant reduction in viral load after incubation with mouse serum (DREP: *p* < 0.01 and VRP: *p* < 0.0001; Figure 5C,D). Consistent with these findings, an immunofluorescence assay of the SARS-CoV-2 S protein confirmed that the antibodies produced in the serum of vaccinated mice could effectively neutralize the SARS-CoV-2 Omicron variant (Figure 5E,F). These findings confirmed the high efficiency of SINV replicon particles in expressing SARS-CoV-2 antigens and inducing immune responses.

### 3.7. Protective Effect of the Combined Vaccine Against DENV and SINV Viruses

To evaluate the neutralizing capacity of sera from mice immunized with the DREP and VRP vaccines against DENV and SINV infections, we preincubated these viruses with the sera and detected viral loads using plaque assays and RT-qPCR. In serum neutralization assays targeting DENV1 infection, all antigen combinations in the VRP vaccine group induced the production of specific antibodies in mice that effectively neutralized DENV1, compared with those in the control group (*p* < 0.05; Figure 5G,H). Similarly, in the DENV2 neutralization assays, sera from mice immunized with all DREP and VRP vaccine groups were effective in neutralizing DENV2 and reducing viral titers compared with those in the control group (*p* < 0.05; Figure 5I,J). For SINV neutralization assays, sera from mice immunized with most of the vaccines demonstrated reduced SINV mRNA levels compared with the control group, which received only PBS, indicating effective viral inhibition (DREP: RBD-DV2-NS1 vs. control, *p* = 0.0229; RBD-DV1-80E vs. control, *p* = 0.0334; HR1HR2-DV2-NS1 vs. control, *p* = 0.0084; HR1HR2-DV2-80E vs. control, *p* = 0.0103; and HR1HR2-DV1-NS1 vs. control, *p* = 0.0074. VRP: *p* < 0.001 (Figure 5K,L). Taken together, these results suggest that sera from mice immunized with these DREP and VRP vaccines exhibited strong neutralization against SARS-CoV-2, DENV, and SINV, confirming that delivering combined vaccines via DREP and VRP is highly feasible.

### 3.8. Evaluation of Vaccine Safety

**Figure 6:**
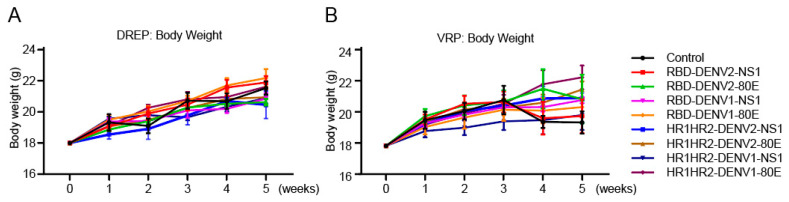
Vaccine safety assessment. (**A**,**B**) The mice were immunized with (**A**) DREP and (**B**) VRP vaccines, and their body weights were measured weekly for 5 weeks, n = 5. Data are presented as mean ± SD.

The body weights of the mice were recorded during the vaccine immunization experiments. Notably, no considerable changes in body weight or adverse signs were observed in the vaccinated groups compared to the controls (Figure 6A,B).

### 3.9. Evaluation of the Protective Effect of the Vaccines Against DENV2 Infection in the AG129 Mouse Model via Passive Immunization

**Figure 7:**
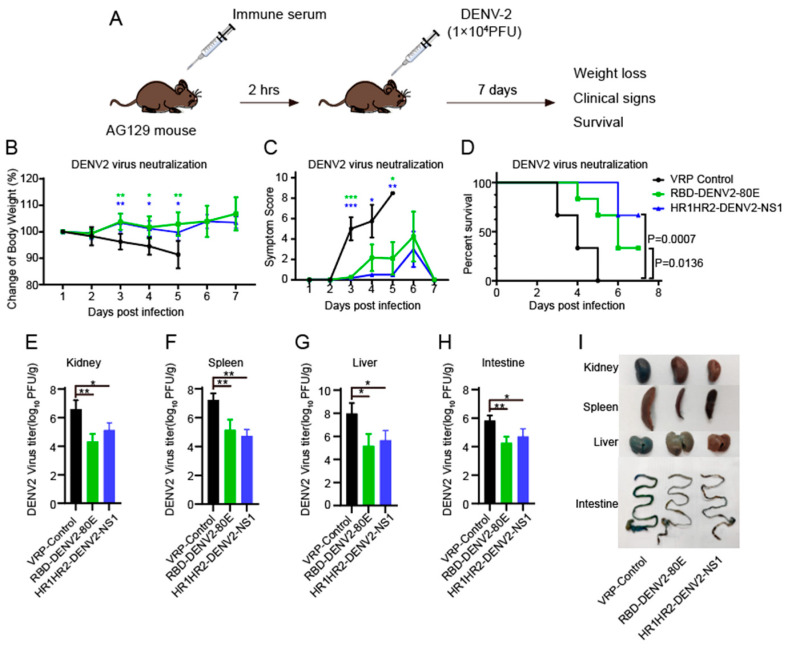
A passive immunization experiment evaluating the protective effect of sera from BALB/c mice immunized with vaccines against the DENV2 challenge in AG129 mice. (**A**) Schematic of the passive immunization experiment in AG129 mice. (**B**–**D**) AG129 mice passively received 100 μL of immune serum from BALB/c mice immunized with VRP vaccine RBD-DENV2-80E or HR1HR2-DENV2-NS1. The control group was passively immunized with serum from BALB/c mice injected with SINV replicon virus particles, followed by the intravenous injection of a sublethal dose of DENV2 (10^4^ PFU/mouse) 2 h later (n = 6). The mice were monitored daily for changes in (**B**) body weight, (**C**) clinical symptoms, (**D**) and survival rate. (**E**–**H**) Plaque assays were performed to evaluate viral titers in the (**E**) kidney, (**F**) spleen, (**G**) liver, and (**H**) intestine of AG129 mice infected with DENV2, n = 3. (**I**) Vascular leakage levels in various tissues of AG129 mice were measured using the Evans blue assay, n = 3. All experimental groups were compared with the control group. Data on daily body weight and clinical symptom scores of the mice were analyzed using a one-way ANOVA, followed by Dunnett’s multiple comparison test (**B**,**C**). Survival analysis was performed using the log-rank (Mantel–Cox) test (**D**). Additional statistical analyses were performed using a one-way ANOVA, followed by Dunnett’s multiple comparison test (**E**–**H**). * *p* < 0.05; ** *p* < 0.01; *** *p* < 0.001.

AG129 mice are known for their susceptibility to dengue due to interferon receptor deficiency. We used the AG129 mouse model to evaluate the protective effect of immune sera against the DENV2 challenge. The mice were administered sera from BALB/c mice immunized with VRP vaccines (RBD-DENV2-80E or HR1HR2-DENV2-NS1). The control group was injected with sera from BABL/c mice that received SINV replicon virus particles, followed by a sublethal DENV2 challenge. Body weight, clinical signs, and survival rates of the mice were recorded over the next 7 days (Figure 7A). We observed significant weight loss, more severe clinical signs, and rapid mortality in the control group. In contrast, the mice that received passive transfer of immune sera withstood the sublethal DENV2 challenge, showing only mild clinical signs (RBD-DENV2-80E vs. control, day 3: *p* = 0.0003; day 4: *p* = 0.0784; day 5: *p* = 0.0117. HR1HR2-DENV2-NS1 vs. control, day 3: *p* = 0.0002; day 4: *p* = 0.0117; day 5: *p* = 0.0025) in the early stages and showing no significant weight loss (RBD-DENV2-80E vs. control, day 3: *p* = 0.0017; day 4: *p* = 0.013; day 5: *p* = 0.0095. HR1HR2-DENV2-NS1 vs. control, day 3: *p* = 0.0027; day 4: *p* = 0.0192; day 5: *p* = 0.0417). Additionally, these mice experienced slower mortality compared to the control group (RBD-DENV2-80E vs. control, *p* = 0.0136; HR1HR2-DENV2-NS1 vs. control, *p* = 0.0007) (Figure 7B–D). On day 3 post-infection, kidney, spleen, liver and intestine samples were collected to assess viral loads and showed significantly lower viral titers in these organs in the passive immunization groups compared to the control group, which were passively immunized with serum from BALB/c mice injected with SINV replicon virus particles (Figure 7E–H). In addition, we assessed vascular leakage using the Evans blue assay. On day 5 post-infection, we found more severe Evans blue leakage in the kidneys, spleen, liver, and intestine of the control group, while the mice that received passive immunization showed only mild leakage (Figure 7I). Taken together, these results demonstrate that sera from the mice immunized with the combined vaccines provided significant protection against DENV2 infection in AG129 mice.

## 4. Discussion

The development of a combined SARS-CoV-2 and DENV vaccine is crucial, considering the current outbreaks of both SARS-CoV-2 and DENV and their high geographical overlap. Notably, a combination of the NVX-CoV2373 vaccine with an influenza vaccine demonstrated similar immune effects to those of either vaccine alone [52]. The success of this simple combination immunization test demonstrates the feasibility of a combination immunization strategy. Wang et al. further optimized the protocol by directly combining the RBD of the SARS-CoV-2 spiking protein, which is commonly used in single-dose vaccine development, with an inactivated influenza A virus to develop a SARS-CoV-2 VLP vaccine with dual protective effects, exhibiting neutralizing activity against both SARS-CoV-2 and H1N1 in an animal study [53]. In the present study, we developed a feasible and effective conjugate vaccine based on the SINV replication subsystem. We selected the RBD region with good immunogenicity as the antigen based on SARS-CoV-2 vaccine studies [54,55]. In addition, previous studies have identified the HR functional domain as a conserved target for vaccine development for current and future SARS-CoV-2 mutants [35,56]. Therefore, to ensure that the combined vaccine has a broad antiviral effect, we added the HR1HR2 functional domain as an antigen and constructed a vaccine by combining RBD and HR1HR2 with the DENV classical antigen NS1 and the more immunogenic 80E. Notably, the combined vaccine could induce high antibody expression post-immunization, with a strong neutralizing effect in mice, thereby effectively protecting against SARS-CoV-2 and DENV2 infections. These findings provide a valuable basis for the further development of efficient combination vaccines aimed at reducing the public health burden of multiviral co-infection.

Novel vaccine platforms that offer safety, efficacy, and the potential for rapid production are important for the development of combined vaccines against these two viral infections. Virus-derived replicon RNA (repRNA) vaccines are mainly delivered through virus-like RNA particles, plasmid DNA, and in vitro-transcribed RNA. repRNA vaccines replicate continuously in host cells and maintain high antigenic expression for a long period of time. Moreover, repRNA vaccines can mimic natural virus infection post-immunization, thus inducing a strong immune response [57]. In recent years, vaccines against various viruses, including RSV, HCV, HIV, SFV, HPV, CHIKV, and SARS-CoV-2, based on viral replicon delivery platforms have been reported [58,59,60]. Among them, repRNA-CoV2 S, a spike protein vaccine against SARS-CoV-2, could effectively activate immune responses and stimulate antibody production in mice [61,62,63]. Furthermore, the VRP-S vaccine could elicit a high antibody response and strong neutralizing activity, along with high levels of neutralizing antibodies for an extended period post-immunization, demonstrating an advantage of the replicon vaccine platform [64,65].

Both the DREP and VRP vaccines in this study efficiently expressed antigens and produced high antibody titers in mice, effectively neutralizing SARS-CoV-2 and DENV2 infections. To optimize each vaccine’s immune response, we employed different immunization methods. VRP vaccines using SFV from the Alphavirus genus have shown that intravenous injection can effectively stimulates immune responses in mice [66,67]. Intravenous injection is further supported by studies on tuberculosis and BCG vaccines, which demonstrate improved immune protection [68]. Following intravenous administration of the VRP vaccine, we observed a strong cellular immune response, with significant upregulation of IFN-γ and TNF-α, and enhanced neutralizing activity. For the DREP plasmid vaccine, we selected intramuscular injection due to its simplicity and efficiency, ensuring high uptake and effective antigen delivery, despite the lower antigen-presenting capacity of muscle tissue [69,70]. Research in multiple species, including humans, shows that VRP vaccines, which resemble viral particles, activate both innate and adaptive immunity, making them a promising platform [71]. This study demonstrates that a combined SINV replicon-based vaccine expressing SARS-CoV-2 and DENV antigens can induce high levels of neutralizing antibodies against both viruses in BALB/c mice, and the passive transfer of immune serum protects AG129 mice from DENV2 infection and DENV2-induced vascular leakage in various tissues. Overall, these findings demonstrate that the combined vaccines are promising candidates for combating COVID-19 and DENV coinfection. This study has certain limitations. We did not evaluate the protective effect of the combined SARS-CoV-2 and DENV vaccine against live SARS-CoV-2 infection in a mouse model. Future studies should utilize mouse models to assess the ability of these vaccines to induce protective immunity against SARS-CoV-2 infection.

## 5. Conclusions

We have developed a combined vaccine targeting SARS-CoV-2 and DENV infections using the SINV replicon system. This potent dual vaccine demonstrated strong immunogenicity and induced antibody production against SARS-CoV-2 and DENV. It holds promise for reducing the number of vaccinations required, easing the burden on healthcare systems, and providing enhanced protection during outbreaks of co-infections.

## Data Availability

Data are contained within the article and Supplementary Material.

## Supplementary Materials

The following supporting information can be downloaded at: https://www.mdpi.com/article/10.3390/vaccines12111292/s1. Supplementary materials include uncropped Western blot images and raw data for all figures and tables.
