# Supplementary material for: Sindbis Virus Replicon-Based SARS-CoV-2 and Dengue Combined Vaccine Candidates Elicit Immune Responses and Provide Protective Immunity in Mice
Source: Vaccines (Basel). 2024 Nov 19;12(11):1292. doi: 10.3390/vaccines12111292 (PMC11599113; doi:10.3390/vaccines12111292)

Fig 2C Flag

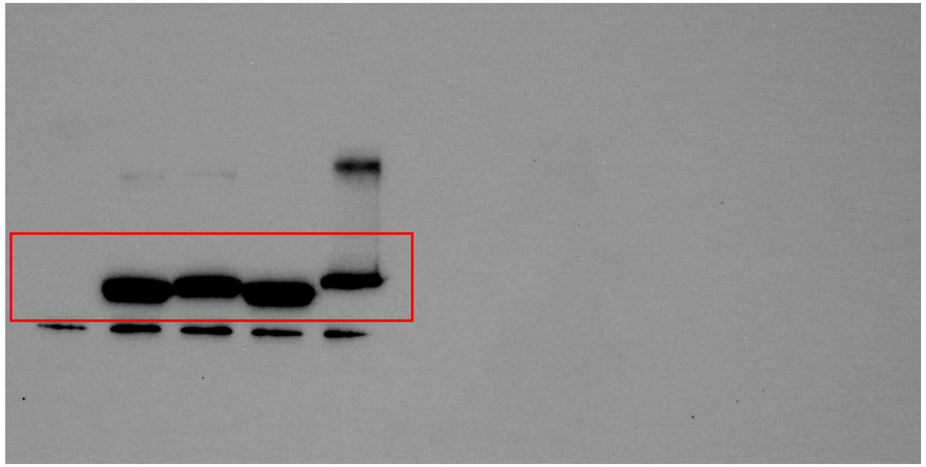

Fig 2C Flag-Membrane

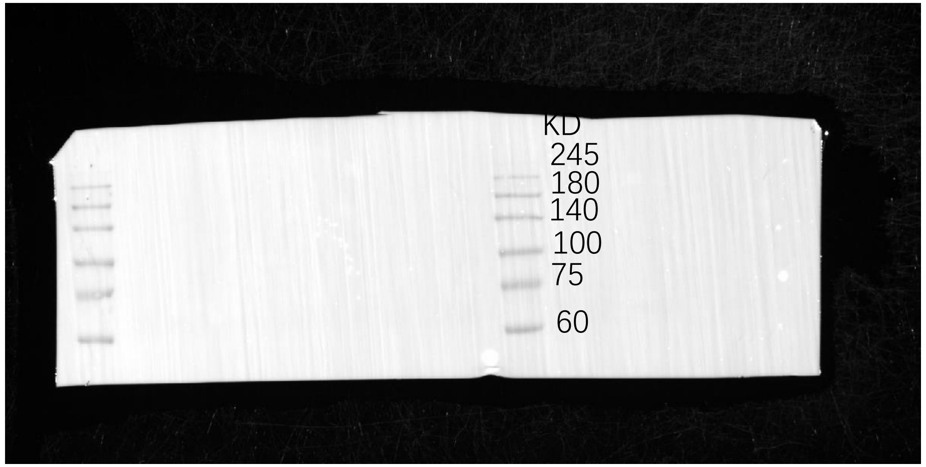

Fig 2C GAPDH

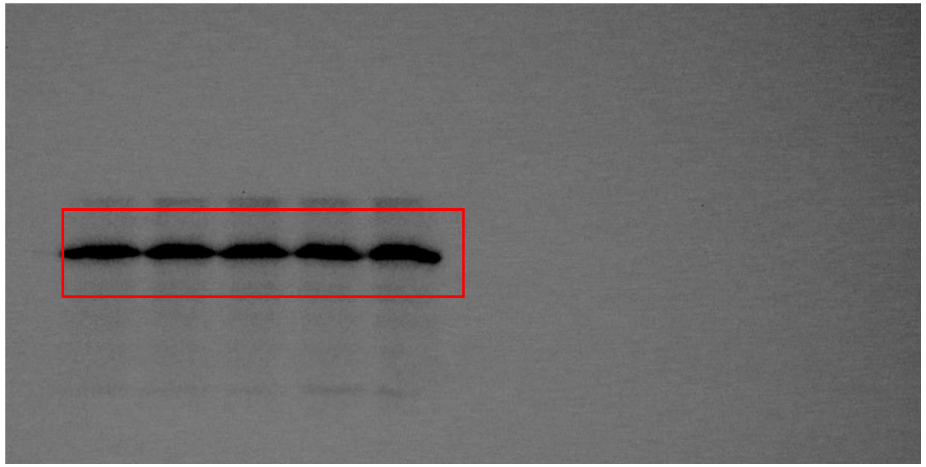

Fig 2C GAPDH-Membrane

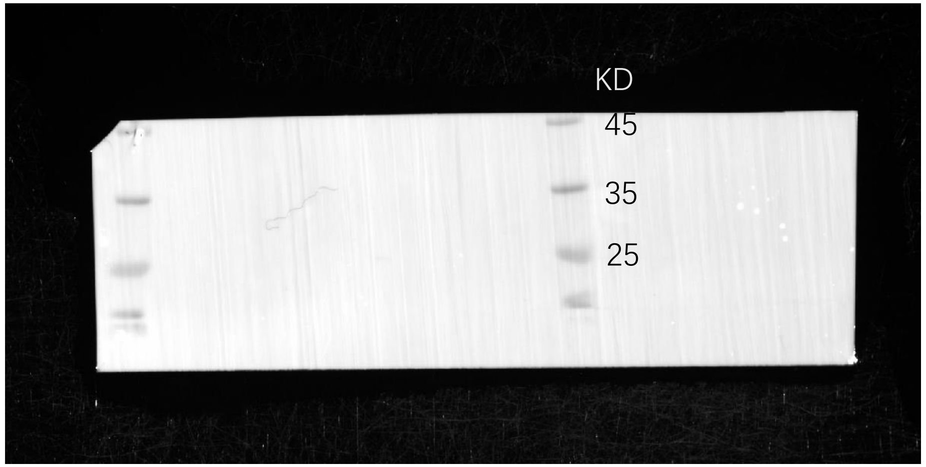

Fig 2D Flag

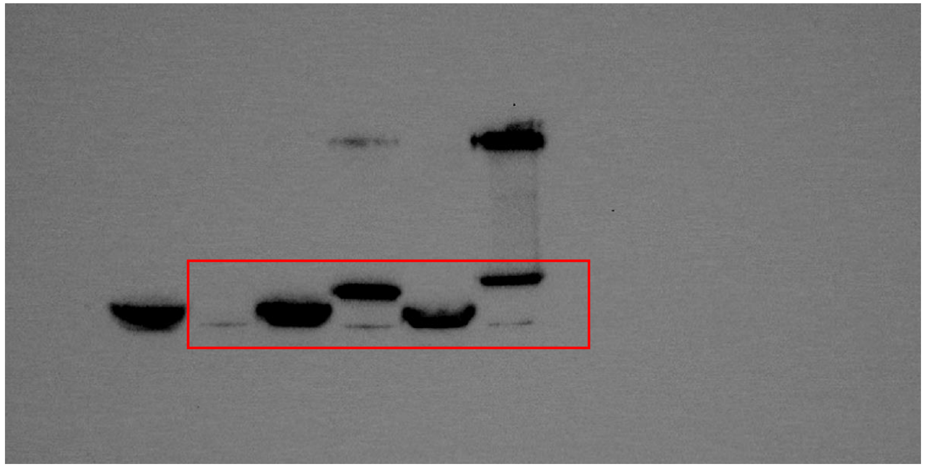

Fig 2D Flag-Membrane

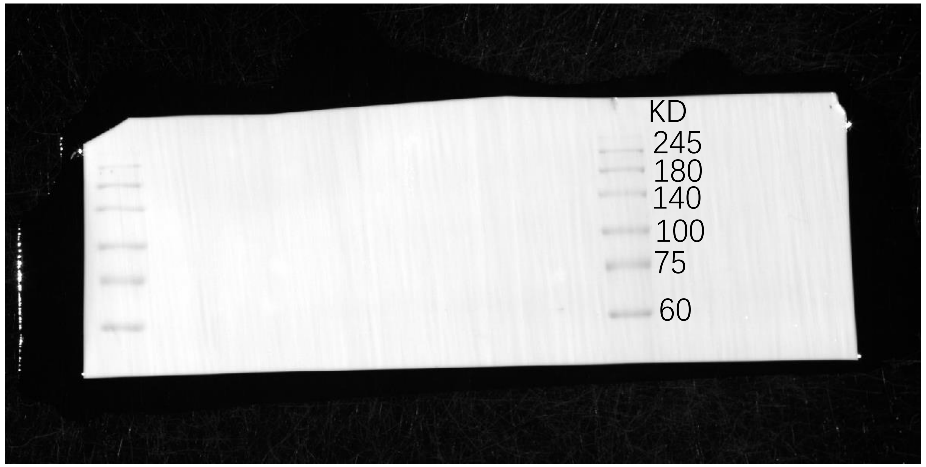

Fig 2D GAPDH

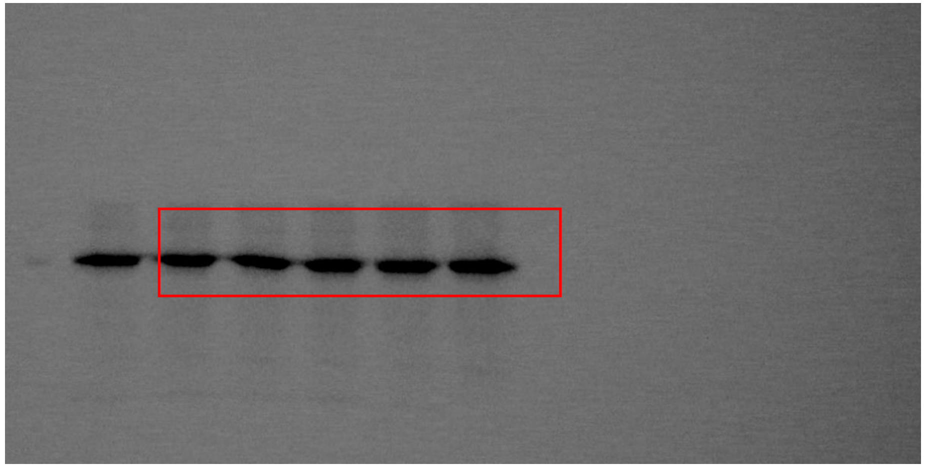

Fig 2D GAPDH-Membrane

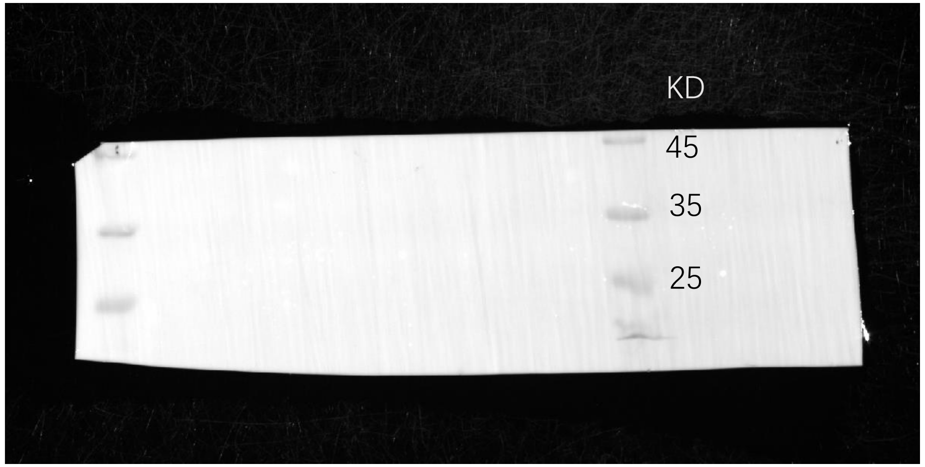

Fig 3E Flag

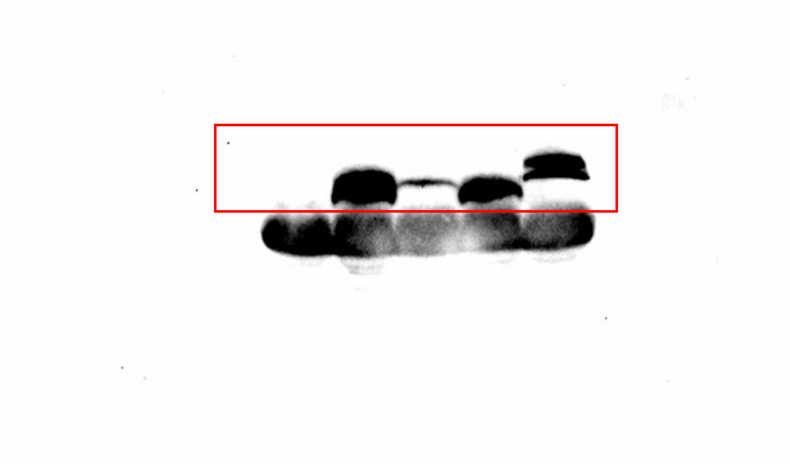

Fig 3E Membrane

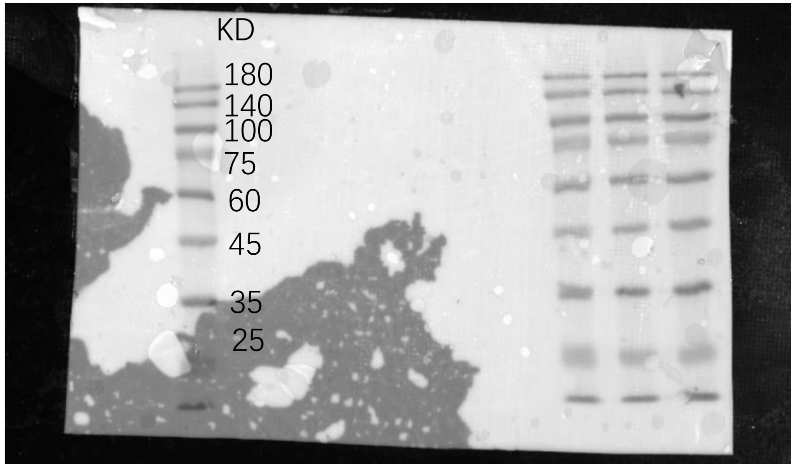

Fig 3F Flag

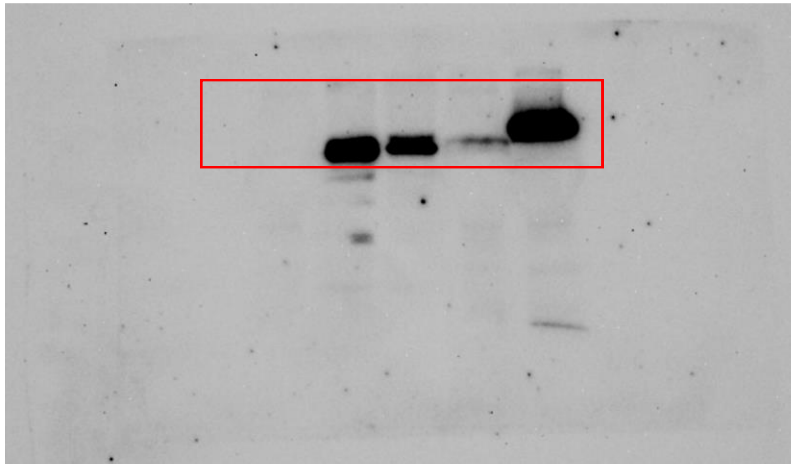

Fig3F Membrane

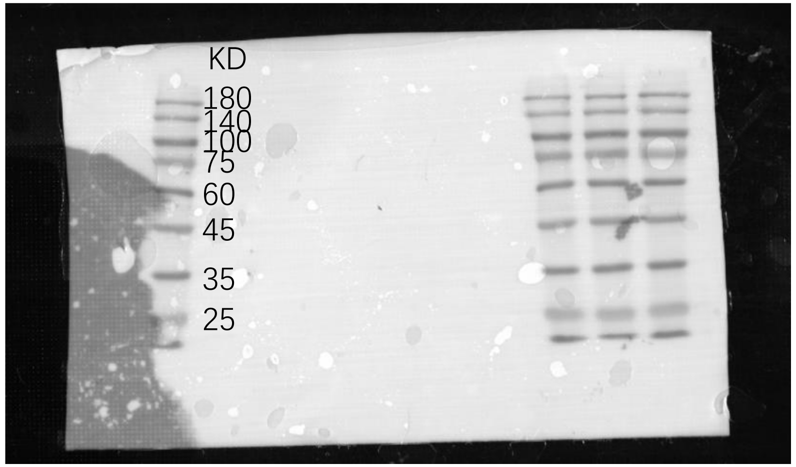

Supplement: Supplementary file 1 [file vaccines-12-01292-s001.zip › Uncropped WB images Fig 2C-D+Fig 3E-F.pdf]
